# A new genus and new species in the tribe Uramyini (Diptera: Tachinidae) from Area de Conservación Guanacaste in northwestern Costa Rica

**DOI:** 10.3897/BDJ.8.e48907

**Published:** 2020-02-21

**Authors:** AJ Fleming, D. Monty Wood, M. Alex Smith, Tanya Dapkey, Winnie Hallwachs, Daniel Janzen

**Affiliations:** 1 Agriculture Agri-Food Canada, Ottawa, Canada Agriculture Agri-Food Canada Ottawa Canada; 2 University of Guelph, Guelph, Canada University of Guelph Guelph Canada; 3 University of Pennsylvania, Philadelphia, United States of America University of Pennsylvania Philadelphia United States of America

## Abstract

**Background:**

We describe one new genus and its one new species from Area de Conservación Guanacaste (ACG) in northwestern Costa Rica. Our study provides a concise description of this new species using morphology, life history, molecular data and photographic documentation.

**New information:**

*Chorotegamyia* **gen. n.** is described, along with its type species, *Chorotegamyia aureofacies* **sp. n.** A modified key to the Uramyini is given to further elucidate the tribe.

## Introduction

The tribe Uramyini was originally proposed by Townsend (1936) as an assemblage of New World genera. This tribal concept *sensu* Townsend was somewhat vague, including many different characters states which, when examined on a broader scale, could be quite variable. The concept, as proposed by Townsend (1936), was later revised in Guimarães (1980). In his work on the Uramyini, Guimarães provided a more concise and concrete definition of the tribe, adding, however, that his concept of the tribe was not a natural assemblage and required much more work. This latter concept was the basis of more recent works, such as Fleming et al. (2015) and Fleming et al. (2017) adding to and building upon the concept as set out by Townsend and improved by Guimarães. Most recently, it has been suggested that the Uramyini are a phylogenetically nested sub-clade within the tribe Voriini (Stireman et al. 2019); this evidence, is still the subject of discussion, so for the sake of continuity and taking into account all the available evidence, the authors hereby propose that the Uramyini continue to be recognized as a tribe within the the Dexiinae, until further examination is conducted to clarify the classification.

This paper proposes a new genus and new species within the tribe Uramyini. The most basic synapomorphies of the Uramyini include the following combination of shared character states: bare prosternum, bare arista, haired eyes, frontal setae extending only to the base of the pedicel, the short fine anepimeral seta, cylindrical bodyplan, the "u"- shaped lappets of the posterior spiracle and the hinged "L"-shaped terminalia with the end of the basiphallus extending beyond the hinge with the distiphallus (Townsend 1936, Guimarães 1980, Fleming et al. 2015, Fleming et al. 2017). Additionally, our new genus also seems to have an affinity for caterpillars within the family Megalopygidae; however, more material would be needed to make facts out of such a conclusion (Arnaud 1978, Guimarães 1980, Wood and Zumbado 2010, Fleming et al. 2015, Fleming et al. 2017).

## Materials and methods

### Voucher specimen management

The management of voucher specimens has been detailed in previous papers in this series, most recently by Fleming et al. 2019. The associated data for each voucher code are available at: http://janzen.bio.upenn.edu/caterpillars/database.lasso. All associated data and successful barcodes are permanently and publicly deposited in the Barcode of Life Data System (BOLD, www.boldsystems.org) (Ratnasingham and Hebert 2007). A select set of these data also subsequently migrated to GenBank. Each barcoded specimen also receives accession numbers from the BOLD and GenBank, respectively. The dynamic nature of the inventory means that it is continually adding new specimens, which can be found by searching for the genus *Chorotegamyia* in BOLD.

All caterpillars reared from the ACG efforts receive a unique voucher code in the format yy–SRNP–xxxxx. Any parasitoid emerging from a caterpillar receives the same voucher code as a record of the rearing event. If and when the parasitoid is later dealt with individually, it receives a second voucher code unique to it, in the format DHJPARxxxxxxx. These voucher codes, assigned to both host and parasitoids, may be used to obtain the individual rearing record at http://janzen.bio.upenn.edu/caterpillars/database.lasso.

All inventoried specimens, discussed herein, were collected under Costa Rican government research permits issued to DHJ and the Tachinidae samples were exported under permit by DHJ from Costa Rica to their final depository in the CNC. Tachinid identifications for the inventory are done by DHJ in coordination with a) visual inspection of morphology by AJF and DMW, b) DNA barcoding by MAS and CBG (Center for Biodiversity Genomics, Guelph) and c) databasing and association with host caterpillars by DHJ and WH through the inventory itself.

The date of capture, cited for each specimen, is the date of eclosion of the fly and not the date of capture of the caterpillar. The eclosion date is much more representative of the time when that fly species is on the wing than is the time of capture of the parasitized caterpillar. The “collector” is the parataxonomist who found the caterpillar, rather than the person who later retrieved the newly eclosed fly and processed it by freezing, pinning, labelling and oven-drying. The primary type material of the newly-described species is housed in the Diptera collection of the Canadian National Collection (CNC).

### Acronyms for depositories

CNC Canadian National Collection of Insects, Arachnids and Nematodes, Ottawa, Canada

### Imaging and dissections

The species account and description, presented in this paper, are complemented by a series of color photos, used to illustrate morphology. Terminology used follows Cumming and Wood (2009). The characters in our description are presented in order of appearance on the body from anterior to posterior and arranged by the headings **Head**, **Thorax**, **Abdomen** and **Terminalia**. All dissections and photography were carried out, following the methods detailed in Fleming et al. (2014). Measurements and examples of anatomical landmarks, discussed herein, are illustrated in Fig. 1.

### DNA Barcoding

We generated DNA extracts from single legs using a standard glass fibre protocol (Ivanova et al. 2006), using the standard DNA barcode region (5’ cytochrome c oxidase I (COI) gene) for all specimens of ACG *Chorotegamyia*. The DNA barcodes (658 bp near the 5’ terminus of the COI gene) were amplified using general insect primers, using standard protocols for both production and quality control (Smith et al. 2006, Smith et al. 2007, Smith et al. 2008, Smith et al. 2009, Smith et al. 2012). Amplicons were evaluated using Sequencher version 5.0 (Gene Codes), examined by eye using Bioedit (Hall 1999) and aligned using Muscle (Edgar 2004). After these quality control checks, all DNA sequences, trace files and accessions were deposited in the Barcode of Life Data System (BOLD) (Ratnasingham and Hebert 2007). Metadata (including GenBank accession codes), associated with each sequence, can be consulted on BOLD, by using the persistent DOI dx.doi.org/10.5883/DS-ASCHOROT.

## Taxon treatments

### Chorotegamyia

Fleming & Wood gen. n.

749D5739-6957-5120-B68C-E8445F61FD13

urn:lsid:zoobank.org:act:0002FF35-10CB-4100-AEE2-66A69D45101F

Chorotegamyia Chorotegamyia aureofacies Fleming & Wood Status: new species described in this paper.

#### Description

**Male**: **Head**: slightly elongate, in frontal view slightly taller than wide, triangular in profile, wider at axis of pedicel than at axis of vibrissa. Height of gena 0.3x-0.4x eye height. Inner vertical setae stout, incurved and medially crossed, nearly half of eye height; outer vertical setae short to absent. Ocellar triangle extending into occiput, ocellar setae strong and proclinate. Fronto-orbital plate with lowest frontal seta situated at level of base of pedicel. Frontal vitta prominent and inwardly pinched, at narrowest point narrower than ocellar triangle. Vibrissae crossed, level with lower facial margin. Facial ridge bare. Antennal insertion situated below middle level of eye. Pedicel with short dorsal setae equal in length to pedicel; postpedicel short and slightly bean-shaped, twice as long as pedicel, short and parallel-sided, rounded at apex. Arista elongate, bare and filiform. Eyes haired. **Thorax**: prosternum bare. Thoracic chaetotaxy: 3:3 acrostichal setae; 3:3 dorsocentral setae; 2:2 intra-alar setae; 2:2 supra-alar setae; 4 postpronotal setae; 3 katepisternal setae, 2 anterior and 1 posterior to suture. Scutellum densely haired, strong pair of medially crossed preapical setae and 3 pairs of marginal setae. One pair of weak but differentiated widely-spaced scutellar discal setae. One strong anepimeral seta stronger and longer than others, but not extending beyond margin of upper calypter. Meron with 6-10 regular setae. Lappets of posterior spiracle appearing as a fringe of hairs of equal length surrounding the spiracle leaving a central "u"-shaped opening. Postmetacoxal bridge unsclerotized. **Legs**: anteroventral surface of forecoxa gold tomentose with several strong setae, ventral surface bare; anterior tibia with a regularly-sized fringe of equally-spaced setae along anterodorsal surface, posterodorsally with two strong setae. Posterior tibia with 3 large strong posteroventral setae, posterodorsal surface with a ragged irregular fringe of 4–5 strong setae. Tarsal claws black, pulvilli elongate subequal to length of tarsal claws. **Wing**: costal spine absent. R_4+5_ at base with 2–3 setulea dorsally and 1–2 setulae ventrally. Bend of vein M obtuse, vein terminating in wing margin. Stub of CuA_1_ terminating in wing margin, 1.9x length of cross-vein dm-cu. **Abdomen**: abdomen elongate, almost 2x as long as wide; mid-dorsal depression of T1+2 reaching to hind margin; median marginal setae on T3 and complete rows of marginal setae on T4–T5 (reduced on T5); T3–T5 each with one pair of median discal setae. **Terminalia**: cercus sharply pointed and strongly tapered, basal section of syncercus 1/3 as long as apical section; strongly curved when viewed laterally; surstylus narrow and scythe-like in lateral view, apices unusually sharp; surstyli angled medially in dorsal view; surstylus 1.6x as long as cercus. Phallus distinctly hinged as in the remainder of the Dexiinae.

**Female**: As in male with the following exceptions: width of head at widest point 4.2 times width of vertex (in frontal view), profile 1.6x wider at axis of pedicel, than at axis of vibrissa, frontal view head height 1.17x head width. Height of gena 0.43x eye height. Two proclinate orbital setae, hind one slightly shorter than anterior. Postpedicel longer than in males nearly 2.4x as long as pedicel. Height of gena longer in females than in males. **Thorax**: Scutellum densely haired, strong pair of inwardly crossed preapical setae and 3 pairs of marginal setae. Meron with 6-10 regular setae. **Legs**: tarsal claws black, pulvilli short approximately 0.5x length of tarsal claws. **Wing**: as in males. **Abdomen**: abdomen elongate but slightly more globose than male; median marginal setae on T3 and complete row of marginal setae on T4–T5; T3–T5 each with one pair of median discal setae. **Terminalia**: not examined.

#### Diagnosis

Our diagnosis of the new genus suggests it belongs to the tribe Uramyini, based on the following combination of character states which it shares with its tribemates (*Uramya* Robineau-Desvoidy, *Itaplectops* Townsend and *Thelairaporia* Guimaraes): frontal setae extending only to the base of the pedicel, bare prosternum, the short fine anepimeral seta not reaching the midpoint of the lower calypter, vein R_4+5_ ending at wing margin, elongated "cylindrical" body plan, the "u"-shaped lappets of the posterior spiracle and the distinctively hinged and "L"-shaped terminalia with the end of the basiphallus extending beyond the hinge with the distiphallus (Townsend 1936, Guimarães 1980, Fleming et al. 2015, Fleming et al. 2017). *Chorotegamyia* can be differentiated from the rest of the Uramyini by the following combination of character states: presence of proclinate orbital setae only in females, short weak vertical setae, two postsutural supra-alar setae, only one pair of discal setae on T3–T5 and mid-dorsal depression on T1+2 extending almost to tergal margin.

#### Etymology

*Chorotegamyia* **gen. n.** is named with reference to the Chorotega Indian tribe historical residents of the province of Guanacaste in North Western Costa Rica.

#### Distribution

Costa Rica, ACG, Guanacaste Province, 295 m elevation.

### Chorotegamyia aureofacies

Fleming & Wood sp. n.

1884995B-4B3B-5FD9-895D-DF79224590DB

urn:lsid:zoobank.org:act:8DFF1145-5B8F-4D8B-9296-7129FB6B39A2

#### Materials

- **Type status:** Holotype. **Occurrence:** occurrenceDetails: http://janzen.sas.upenn.edu; catalogNumber: DHJPAR0040802; recordedBy: D.H. Janzen, W. Hallwachs & Dunia Garcia; individualID: DHJPAR0040802; individualCount: 1; sex: M; lifeStage: adult; preparations: pinned; otherCatalogNumbers: ASHYE2938-11, 09-SRNP-15573, BOLD:AAT8882; **Taxon:** scientificName: Chorotegamyia aureofacies; phylum: Arthropoda; class: Insecta; order: Diptera; family: Tachinidae; genus: Chorotegamyia; specificEpithet: aureofacies; scientificNameAuthorship: Fleming & Wood, 2019; **Location:** continent: Central America; country: Costa Rica; countryCode: CR; stateProvince: Guanacaste; county: Sector Santa Rosa; locality: Area de Conservacion Guanacaste; verbatimLocality: Area Administrativa; verbatimElevation: 295; verbatimLatitude: 10.8376; verbatimLongitude: -85.6187; verbatimCoordinateSystem: Decimal; decimalLatitude: 10.8376; decimalLongitude: -85.6187; **Identification:** identifiedBy: AJ Fleming; dateIdentified: 2019; **Event:** samplingProtocol: Reared from the pupa of the Megalopygidae, *Norape nigrovenosa*; verbatimEventDate: 10-Sep-2010; **Record Level:** language: en; institutionCode: CNC; collectionCode: Insects; basisOfRecord: Pinned Specimen
- **Type status:** Paratype. **Occurrence:** occurrenceDetails: http://janzen.sas.upenn.edu; catalogNumber: DHJPAR0040794; recordedBy: D.H. Janzen, W. Hallwachs & Dunia Garcia; individualID: DHJPAR0040794; individualCount: 1; sex: F; lifeStage: adult; preparations: pinned; otherCatalogNumbers: ASHYE2930-11, 09-SRNP-15484, BOLD:AAT8882; **Taxon:** scientificName: Chorotegamyia aureofacies; phylum: Arthropoda; class: Insecta; order: Diptera; family: Tachinidae; genus: Chorotegamyia; specificEpithet: aureofacies; scientificNameAuthorship: Fleming & Wood, 2019; **Location:** continent: Central America; country: Costa Rica; countryCode: CR; stateProvince: Guanacaste; county: Sector Santa Rosa; locality: Area de Conservacion Guanacaste; verbatimLocality: Area Administrativa; verbatimElevation: 295; verbatimLatitude: 10.8376; verbatimLongitude: -85.6187; verbatimCoordinateSystem: Decimal; decimalLatitude: 10.8376; decimalLongitude: -85.6187; **Identification:** identifiedBy: AJ Fleming; dateIdentified: 2019; **Event:** samplingProtocol: Reared from the pupa of the Megalopygidae, *Norape nigrovenosa*; verbatimEventDate: 02-Oct-2010; **Record Level:** language: en; institutionCode: CNC; collectionCode: Insects; basisOfRecord: Pinned Specimen
- **Type status:** Paratype. **Occurrence:** occurrenceDetails: http://janzen.sas.upenn.edu; catalogNumber: DHJPAR0040795; recordedBy: D.H. Janzen, W. Hallwachs & Dunia Garcia; individualID: DHJPAR0040795; individualCount: 1; sex: M; lifeStage: adult; preparations: pinned; otherCatalogNumbers: ASHYE2931-11, 09-SRNP-15516, BOLD:AAT8882; **Taxon:** scientificName: Chorotegamyia aureofacies; phylum: Arthropoda; class: Insecta; order: Diptera; family: Tachinidae; genus: Chorotegamyia; specificEpithet: aureofacies; scientificNameAuthorship: Fleming & Wood, 2019; **Location:** continent: Central America; country: Costa Rica; countryCode: CR; stateProvince: Guanacaste; county: Sector Santa Rosa; locality: Area de Conservacion Guanacaste; verbatimLocality: Area Administrativa; verbatimElevation: 295; verbatimLatitude: 10.8376; verbatimLongitude: -85.6187; verbatimCoordinateSystem: Decimal; decimalLatitude: 10.8376; decimalLongitude: -85.6187; **Identification:** identifiedBy: AJ Fleming; dateIdentified: 2019; **Event:** samplingProtocol: Reared from the pupa of the Megalopygidae, *Norape nigrovenosa*; verbatimEventDate: 28-Sep-2010; **Record Level:** language: en; institutionCode: CNC; collectionCode: Insects; basisOfRecord: Pinned Specimen
- **Type status:** Paratype. **Occurrence:** occurrenceDetails: http://janzen.sas.upenn.edu; catalogNumber: DHJPAR0040796; recordedBy: D.H. Janzen, W. Hallwachs & Dunia Garcia; individualID: DHJPAR0040796; individualCount: 1; sex: F; lifeStage: adult; preparations: pinned; otherCatalogNumbers: ASHYE2932-11, 09-SRNP-15497, BOLD:AAT8882; **Taxon:** scientificName: Chorotegamyia aureofacies; phylum: Arthropoda; class: Insecta; order: Diptera; family: Tachinidae; genus: Chorotegamyia; specificEpithet: aureofacies; scientificNameAuthorship: Fleming & Wood, 2019; **Location:** continent: Central America; country: Costa Rica; countryCode: CR; stateProvince: Guanacaste; county: Sector Santa Rosa; locality: Area de Conservacion Guanacaste; verbatimLocality: Area Administrativa; verbatimElevation: 295; verbatimLatitude: 10.8376; verbatimLongitude: -85.6187; verbatimCoordinateSystem: Decimal; decimalLatitude: 10.8376; decimalLongitude: -85.6187; **Identification:** identifiedBy: AJ Fleming; dateIdentified: 2019; **Event:** samplingProtocol: Reared from the pupa of the Megalopygidae, *Norape nigrovenosa*; verbatimEventDate: 28-Sep-2010; **Record Level:** language: en; institutionCode: CNC; collectionCode: Insects; basisOfRecord: Pinned Specimen
- **Type status:** Paratype. **Occurrence:** occurrenceDetails: http://janzen.sas.upenn.edu; catalogNumber: DHJPAR0040797; recordedBy: D.H. Janzen, W. Hallwachs & Dunia Garcia; individualID: DHJPAR0040797; individualCount: 1; sex: M; lifeStage: adult; preparations: pinned; otherCatalogNumbers: ASHYE2933-11, 09-SRNP-15552, BOLD:AAT8882; **Taxon:** scientificName: Chorotegamyia aureofacies; phylum: Arthropoda; class: Insecta; order: Diptera; family: Tachinidae; genus: Chorotegamyia; specificEpithet: aureofacies; scientificNameAuthorship: Fleming & Wood, 2019; **Location:** continent: Central America; country: Costa Rica; countryCode: CR; stateProvince: Guanacaste; county: Sector Santa Rosa; locality: Area de Conservacion Guanacaste; verbatimLocality: Area Administrativa; verbatimElevation: 295; verbatimLatitude: 10.8376; verbatimLongitude: -85.6187; verbatimCoordinateSystem: Decimal; decimalLatitude: 10.8376; decimalLongitude: -85.6187; **Identification:** identifiedBy: AJ Fleming; dateIdentified: 2019; **Event:** samplingProtocol: Reared from the pupa of the Megalopygidae, *Norape nigrovenosa*; verbatimEventDate: 24-Sep-2010; **Record Level:** language: en; institutionCode: CNC; collectionCode: Insects; basisOfRecord: Pinned Specimen
- **Type status:** Paratype. **Occurrence:** occurrenceDetails: http://janzen.sas.upenn.edu; catalogNumber: DHJPAR0040804; recordedBy: D.H. Janzen, W. Hallwachs & Dunia Garcia; individualID: DHJPAR0040804; individualCount: 1; sex: F; lifeStage: adult; preparations: pinned; otherCatalogNumbers: ASHYE2940-11, 09-SRNP-15487, BOLD:AAT8882; **Taxon:** scientificName: Chorotegamyia aureofacies; phylum: Arthropoda; class: Insecta; order: Diptera; family: Tachinidae; genus: Chorotegamyia; specificEpithet: aureofacies; scientificNameAuthorship: Fleming & Wood, 2019; **Location:** continent: Central America; country: Costa Rica; countryCode: CR; stateProvince: Guanacaste; county: Sector Santa Rosa; locality: Area de Conservacion Guanacaste; verbatimLocality: Area Administrativa; verbatimElevation: 295; verbatimLatitude: 10.8376; verbatimLongitude: -85.6187; verbatimCoordinateSystem: Decimal; decimalLatitude: 10.8376; decimalLongitude: -85.6187; **Identification:** identifiedBy: AJ Fleming; dateIdentified: 2019; **Event:** samplingProtocol: Reared from the pupa of the Megalopygidae, *Norape nigrovenosa*; verbatimEventDate: 10-Sep-2010; **Record Level:** language: en; institutionCode: CNC; collectionCode: Insects; basisOfRecord: Pinned Specimen

#### Description

**Male**, length: 11–12 mm (Fig. 2). **Head** (Fig. 2a, b): subtriangular in profile, width of head in frontal view at widest point 4.2x width of vertex, in profile 1.44x wider at axis of pedicel than at axis of vibrissa; head height in frontal view 1.1x head width. Fronto-orbital plate and parafacial uniformly colored deep rich gold, extending to post-occiput, with a single row of medioclinate frontal setae, with a few short black setae interspersed throughout, lowest frontal seta level with base of pedicel; inner vertical setae strong, incurved and medially crossed, 0.4x height of eye; outer vertical setae short near absent. Fronto-orbital plate strongly tapered, at vertex 0.33x as wide as at base of antenna. Ocellar triangle gold tomentose, terminating dorsally in a sharp triangle in occiput; ocellar setae strong and proclinate. Base of antennae situated below middle of eye. Pedicel brilliant orange, with short dorsal setae of vaguely equal length to pedicel; postpedicel short and slightly bean-shaped, nearly 2x as long as pedicel, short and parallel-sided, rounded at apex. Arista elongate, bare and filiform, basally orange and slightly thickened. Parafacial gold, bare; gena 0.3x eye height, haired; facial ridge bare. Vibrissae crossed, level with facial margin. Occiput of head slightly convex, occipital setulae black along outer margins, yellow medially. Palpus short yellow and digitiform with few sparse setulae throughout. **Thorax** (Fig. 2c, d): disc of thorax dark grey almost black, marginally (outside of intra-alar row of setae) with golden tomentosity; lateral view of thorax grey tomentose with gold accents. Thoracic chaetotaxy: 3:3 acrostichal setae; 3:3 dorsocentral setae; 2:2 intra-alar setae; 2:2 supra-alar setae; 4–5 postpronotal setae; 3 katepisternal setae (two anterior and one posterior to suture). Scutellum densely haired, strong pair of inwardly crossed preapical setae and 3 pairs of marginal setae. **Legs** (Fig. 2d): black ground color, tibiae yellow ground color, but densely hirsute so as to appear black; foreleg, coxa with golden tomentum along anterior surface laden with several strong setae. **Wing** (Fig. 2c): slightly infuscate along costal margin extending to and including R_4+5_, brown veins, costal spine absent; basicosta dark brown; calypters pale yellow translucent, marginally setulose, upper calypter ~1.5x as large as lower calypter. **Abdomen** (Fig. 2c, d): abdomen elongate, 2x as long as wide; ST1+2 black, T3 brown tomentose, T4–T5 golden tomentose, with a moderate vestiture of short decumbent black setae; T4 with a mid-dorsal darkened stripe reaching a darkened band along tergal margin occupying 0.16x of tergite; T5 entirely gold reaching apex; mid-dorsal depression of T1+2 reaching to hind margin; median marginal setae on T3 and complete row of marginal setae on T4, marginal setae irregular on T5 medial pair not reaching tergal margin, making their appearance confused as medial discal setae; T3–T4 each with one distinct pair of short median discal setae. **Terminalia** (Fig. 3): inner margin of sternite 5 pollinose, appearing slightly darker than surrounding cuticle; posterior lobes of sternite with short, stout setae interspersed with 5–8 longer setae closer to apical margins; wide V-shaped median cleft, 0.5x length of sternite from lobe apex to base (Fig. 3d). Cercus sharply pointed and strongly tapered, basal section of syncercus 0.4x as long as apical section; strongly curved when viewed laterally and with a sharp upward hook at its tip (Fig. 3b). Surstylus narrow and scythe-like in lateral view, apices sharp; both surstyli and syncercus heavily sclerotized, appearing black despite clearing; surstyli angled inwards in dorsal view (Fig. 3a); almost parallel; surstylus 1.6x as long as cercus. Phallus distinctly hinged as in the remainder of the Dexiinae; dorsal process of basiphallus extending beyond joint with distiphallus. Postgonite elongate, extending well beyond hinge of distiphallus (Fig. 3c).

**Female**, length: 12–13 mm (Fig. 4). **Head** (Fig. 4a, b): as in males with the following exceptions: width of head at widest point 4.2x width of vertex (in frontal view), profile 1.6x wider at axis of pedicel, than at axis of vibrissa, frontal view head height 1.17x head width. Height of gena 0.43x eye height. Fronto-orbital plate with 1–2 pairs of proclinate orbital setae and one hindmost pair of reclinate orbital seta, hind proclinate orbital seta slightly shorter than anterior; sparsely setulose with two sparse rows of setulae outside of frontal setae; not tapered, at vertex subequal to width at base of antenna. Postpedicel short and slightly bean-shaped, slightly longer than in male, nearly 2.4x as long as pedicel. Arista basally brown and slightly thickened. Height of gena 0.45x height of eye. Palpus short yellow and slightly spathulate with few sparse setulae throughout. **Thorax** (Fig. 4c, d): disc of thorax covered in light golden tomentum with the exception of 4 wide dorsal stripes, inner pair reaching midway to second postsutural intra-alar. Thoracic chaetotaxy as in males. **Legs**: as in males. **Wing**: as in males. **Abdomen** (Fig. 4c, d): dark bronze pollinose on T3 and T4, T5 entirely golden pollinose, whole abdomen significantly darker than male; mid-dorsal dark stripe evident on T3–T5; darkened band on both T3 and T4 occupying 0.16 of the tergite along the posterior margin; in lateral view, T3–T4 silver pollinose; abdomen elongate but slightly more globose than male, 1.5x as long as wide; median marginal setae on T3 and complete row of marginal setae on T4–T5; T3–T5 each with one pair of median discal setae. **Terminalia**: not examined.

#### Diagnosis

**Figure 5.**
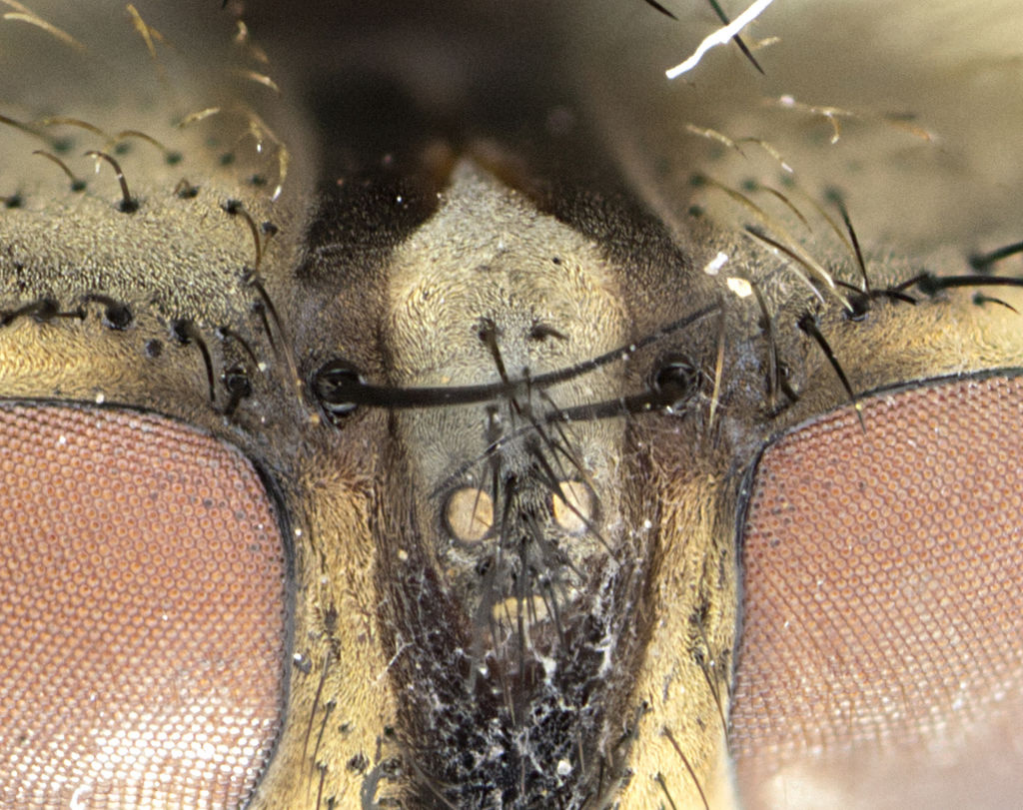
Occipital view of *Chorotegamyia aureofacies* sp. n. highlighting distinctive gold ocellar triangle reaching posteriorly into occiput; image from male holotype.

*Chorotegamyia aureofacies* **sp. n.** is a medium-sized reddish-gold fly. It can be recognized easily within the tribe by the brilliant gold coloration of the fronto-orbital plate and parafacials, the two postsutural supra-alar setae and the distinctive gold ocellar triangle (Fig. 5).

#### Etymology

*Chorotegamyia aureofacies* sp. n. from the Latin adjective, “*aurus*” for gold and the noun "*facies*" for face, with reference to its brilliant gold tomentose head.

#### Distribution

Costa Rica, ACG, Guanacaste Province, 295 m elevation.

#### Ecology

*Chorotegamyia aureofacies* sp. n. has been reared nine times from a single species of Lepidoptera, collected in old secondary succession dry forest, from a massive outbreak of caterpillars of *Norape nigrovenosa* (Druce, 1906) (Megalopygidae). This is the only record of this species of fly out of 4,113 rearings of megalopygid caterpillars in the ACG inventory (1978-2019), of at least 20 species of caterpillars from all ACG ecosystems, 180 of these caterpillars being parasitized by Tachinidae of at least 23 species. This species of caterpillar burrows deep into the soil to spin a very tough double-walled cocoon and the fly larva emerges from the prepupal cadaver to make its puparium inside the moth cocoon next to the cadaver. The batch of caterpillars that yielded the nine *C. aureofacies* specimens (one per caterpillar) was also parasitized by three other species of Tachinidae (*Lespesia*, *Avibrissosturmia*, and *Hyphantrophaga*).

## Identification Keys

### Revised key to the Uramyini

**Table:** 

1	Proclinate orbital setae present only in females; mid-dorsal depression on T1+2 extending almost to tergal margin	2
–	Two strong proclinate orbital setae present in both sexes; mid-dorsal depression on T1+2 not extending to tergal margin	3
2	Vertical bristles of male short weak and proclinate; eyes of male closely approximated medially, nearly obliterating frontal vitta; three postsutural supra-alar setae; often with two or more discal setae on T3–T5	*Uramya* Robineau-Desvoidy
–	Veritcal bristles of male strong and medially crossed; eyes of male not so closely approximated as to obliterate frontal vitta; two postsutural supra-alar setae; only one pair of discal setae on T3–T5	*Chorotegamyia* Fleming & Wood **gen. n.**
3	Facial ridge with four to five small, erect setae above vibrissa; scutellum with pair of widely-separated discal bristles; two katepisternal setae; abdominal tergites lacking discal bristles on T4	*Itaplectops* Townsend
–	Facial ridge with few recumbent hairs above the vibrissa; scutellum without discal setae; three katepisternal setae; abdominal tergites T3 and T4 each with pair of median discal setae	*Thelairaporia* Guimarães

This key is an adaptation of the key to the genera printed in Wood and Zumbado (2010) with some modifications already cited in Fleming et al. (2017). This key should replace Couplets 239–240 in Wood and Zumbado (2010).

## Supplementary Material

XML Treatment for Chorotegamyia

XML Treatment for Chorotegamyia aureofacies

## Figures and Tables

**Figure 1a.**
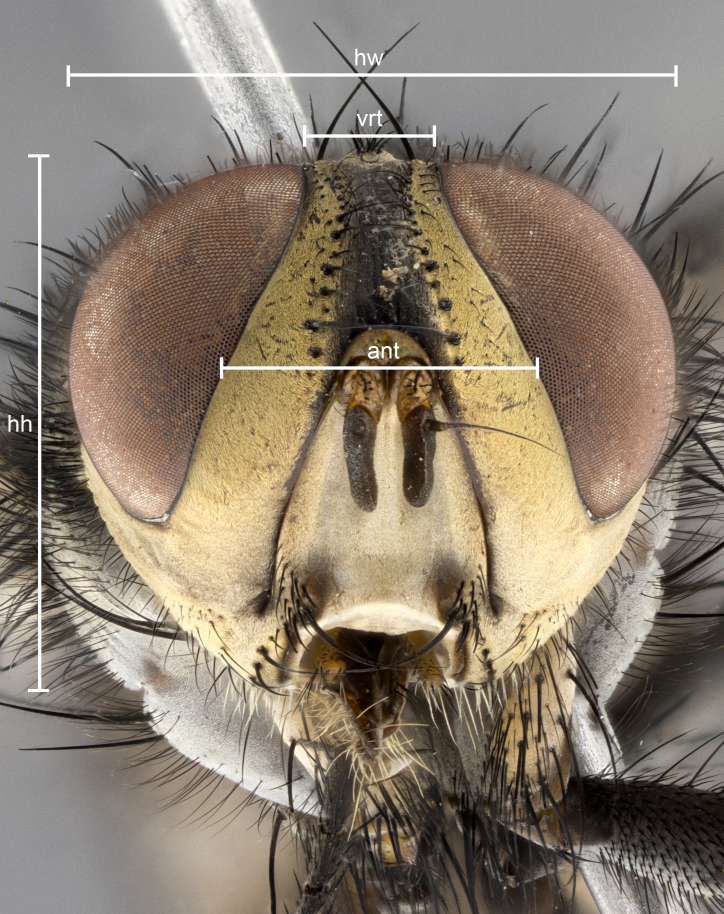
Sample of measured areas from front of head; abbreviations: ant, axis of antenna; hh, head height; hw, head width; vrt, axis of vertex.

**Figure 1b.**
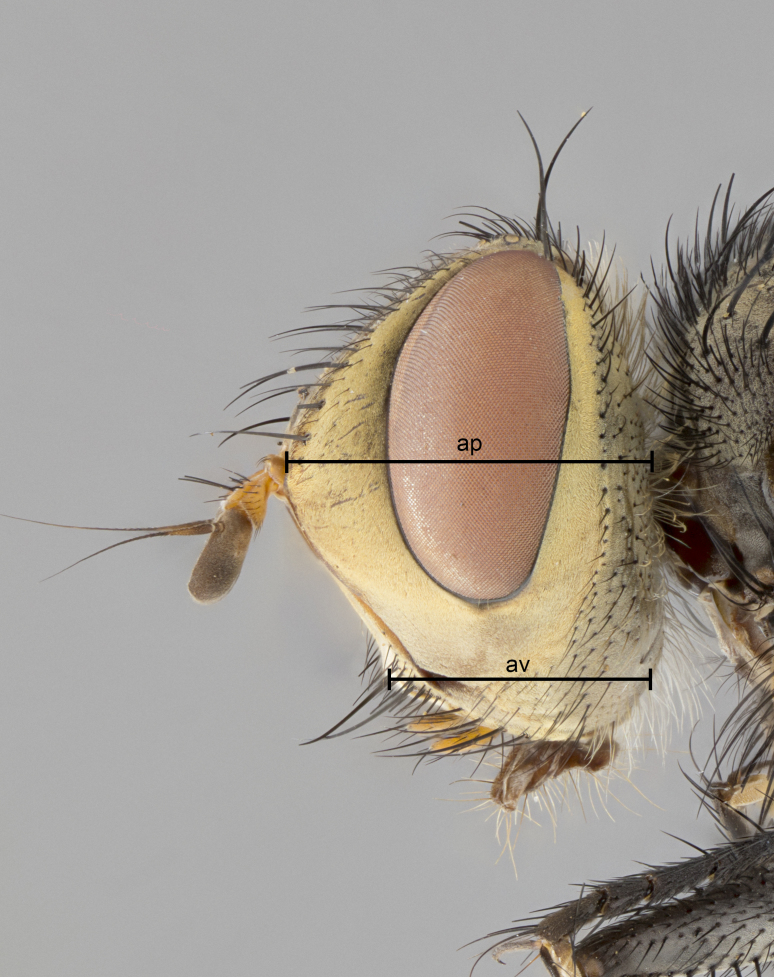
Sample of measured areas from profile of head; abbreviations: ap, axis of pedicel; av, axis of vibrissa.

**Figure 1c.**
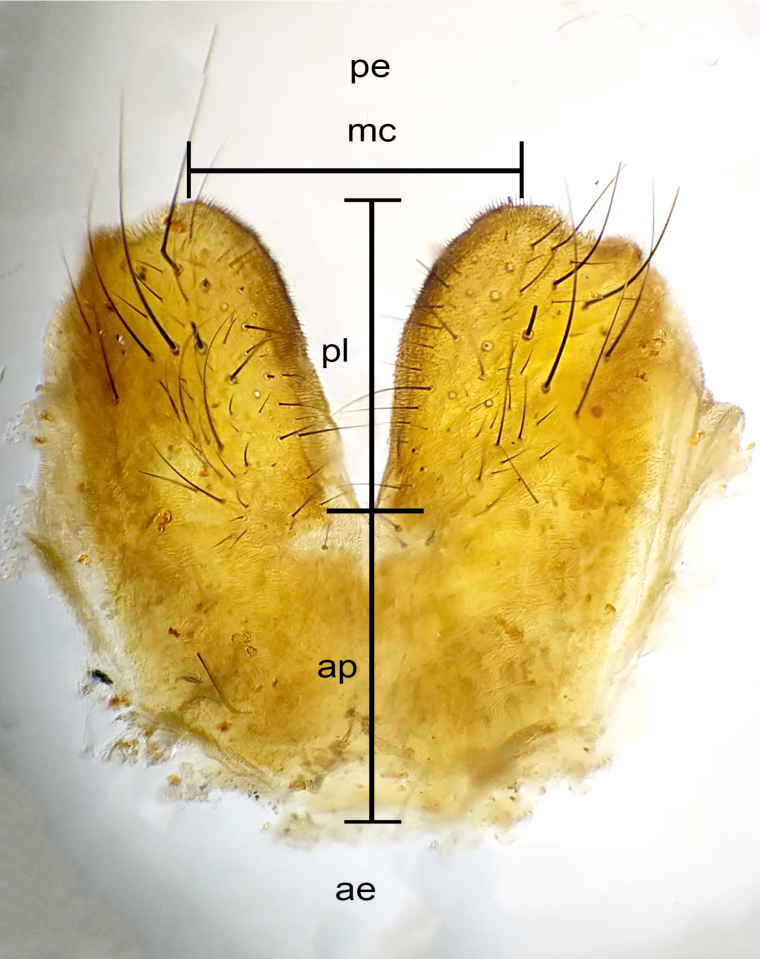


**Figure 1d.**
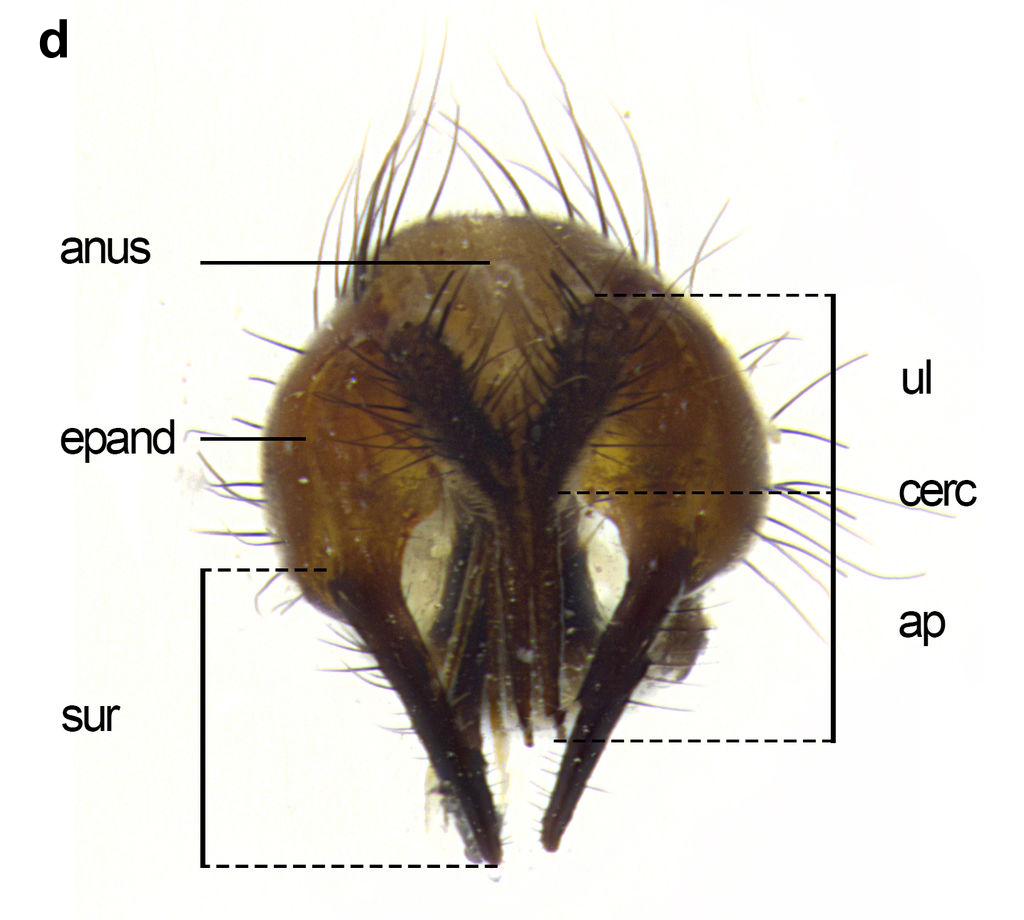


**Figure 2a.**
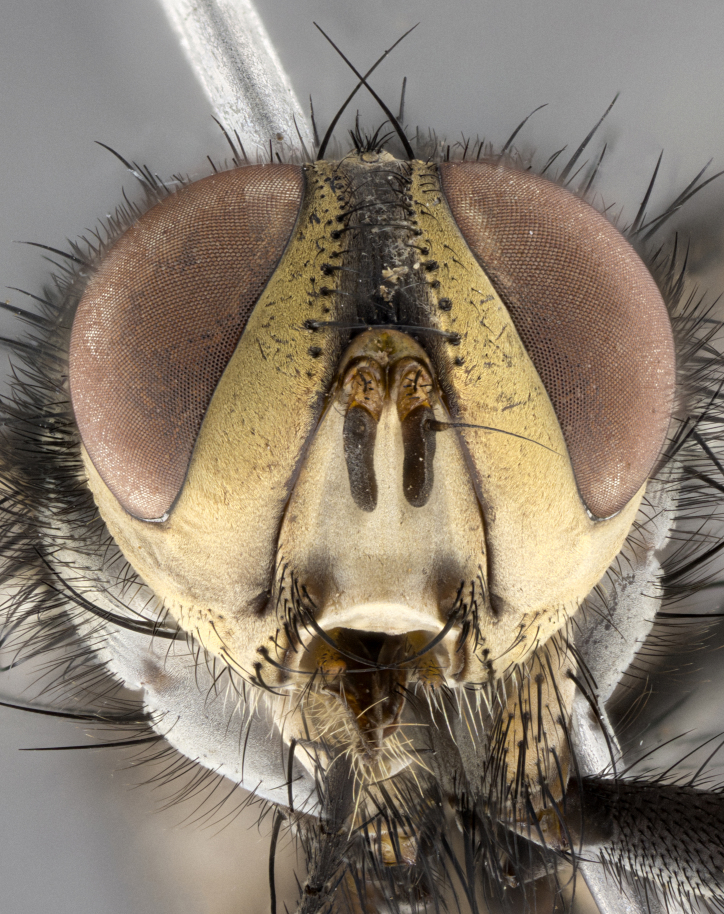
frontal view of head

**Figure 2b.**
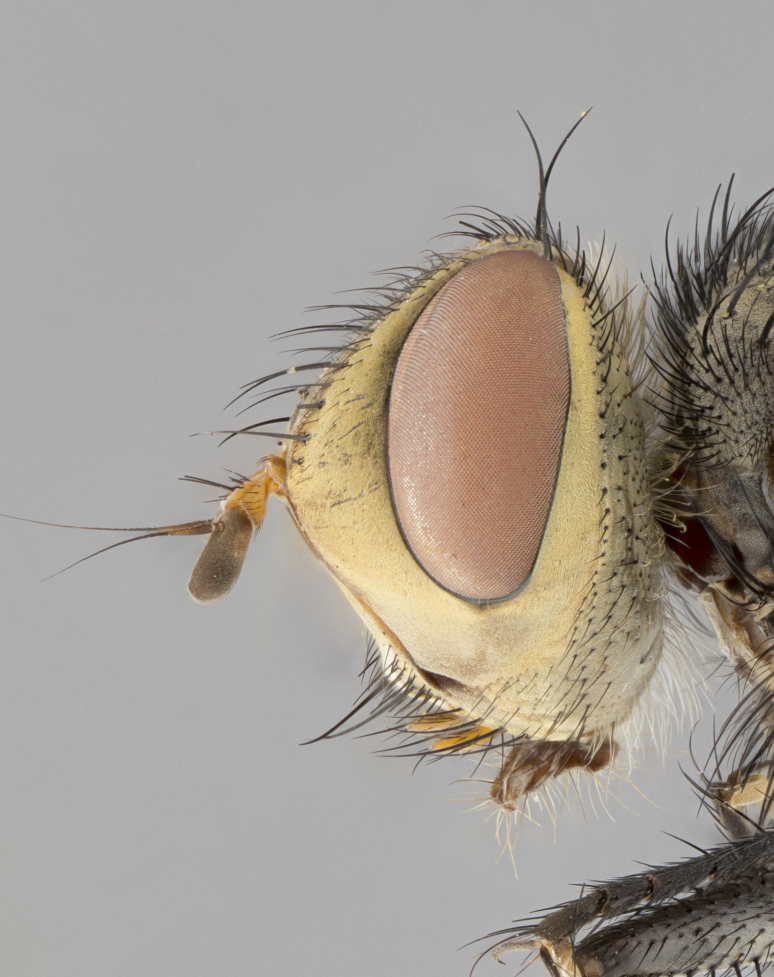
lateral view of head

**Figure 2c.**
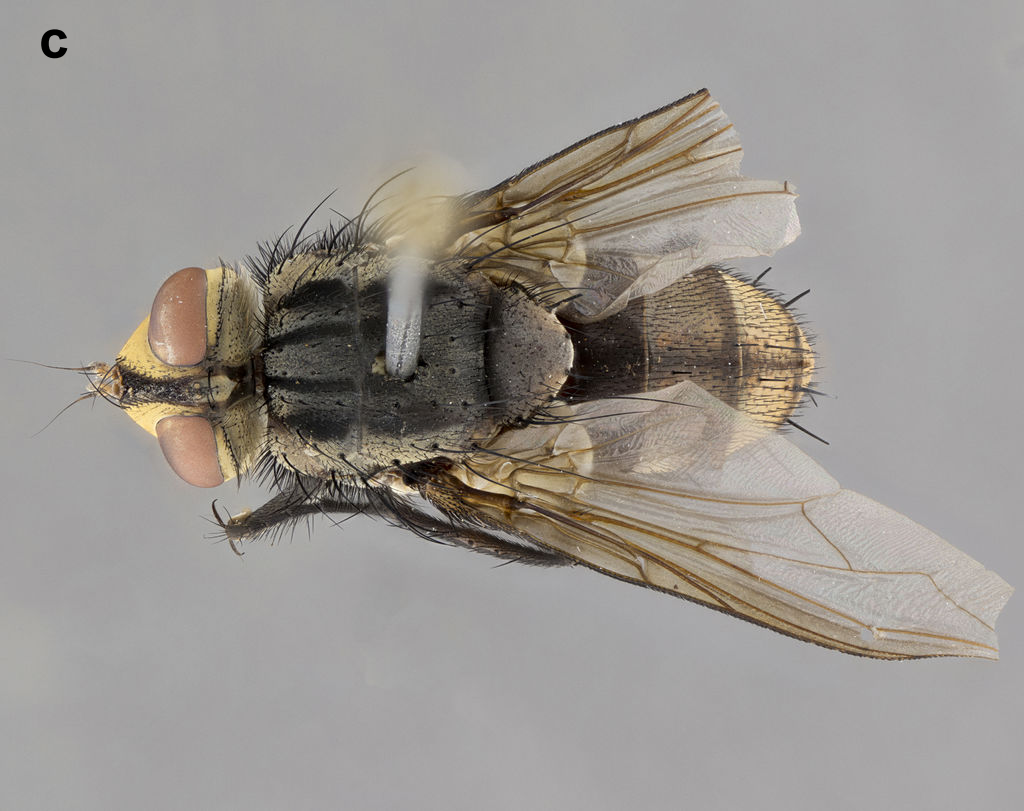
dorsal view

**Figure 2d.**
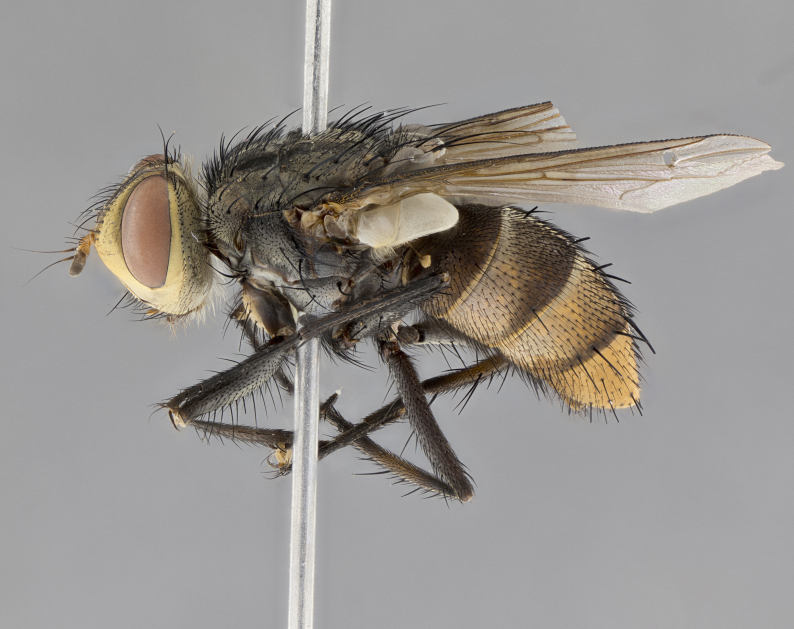
lateral view

**Figure 3a.**
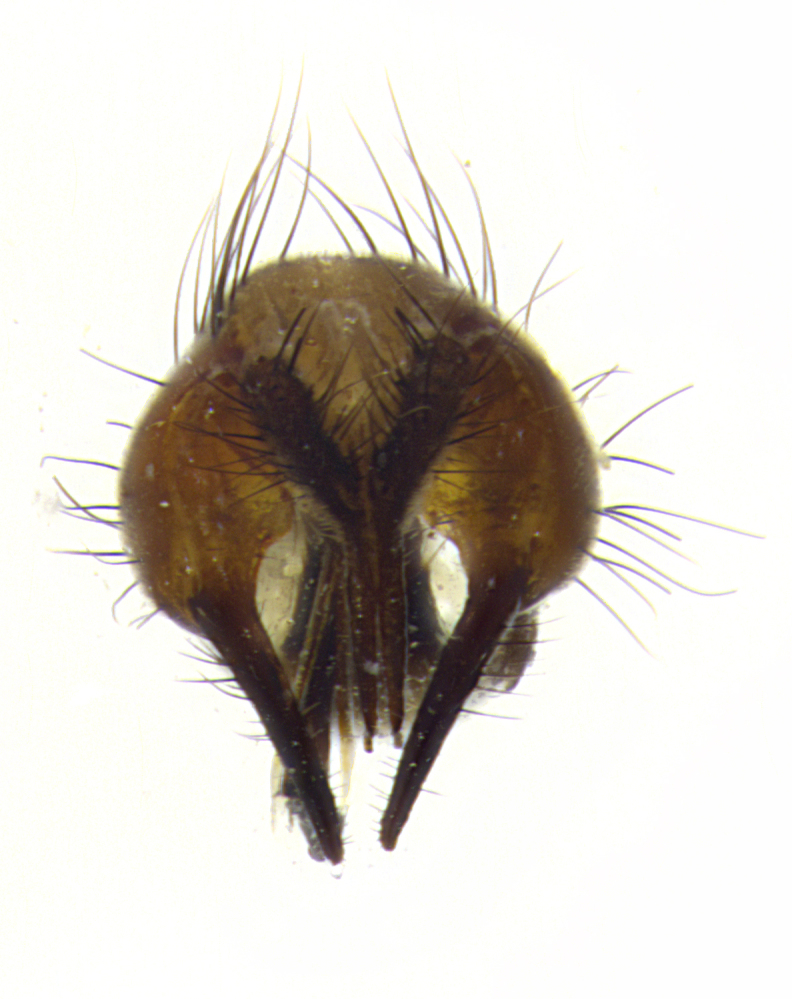
caudal view

**Figure 3b.**
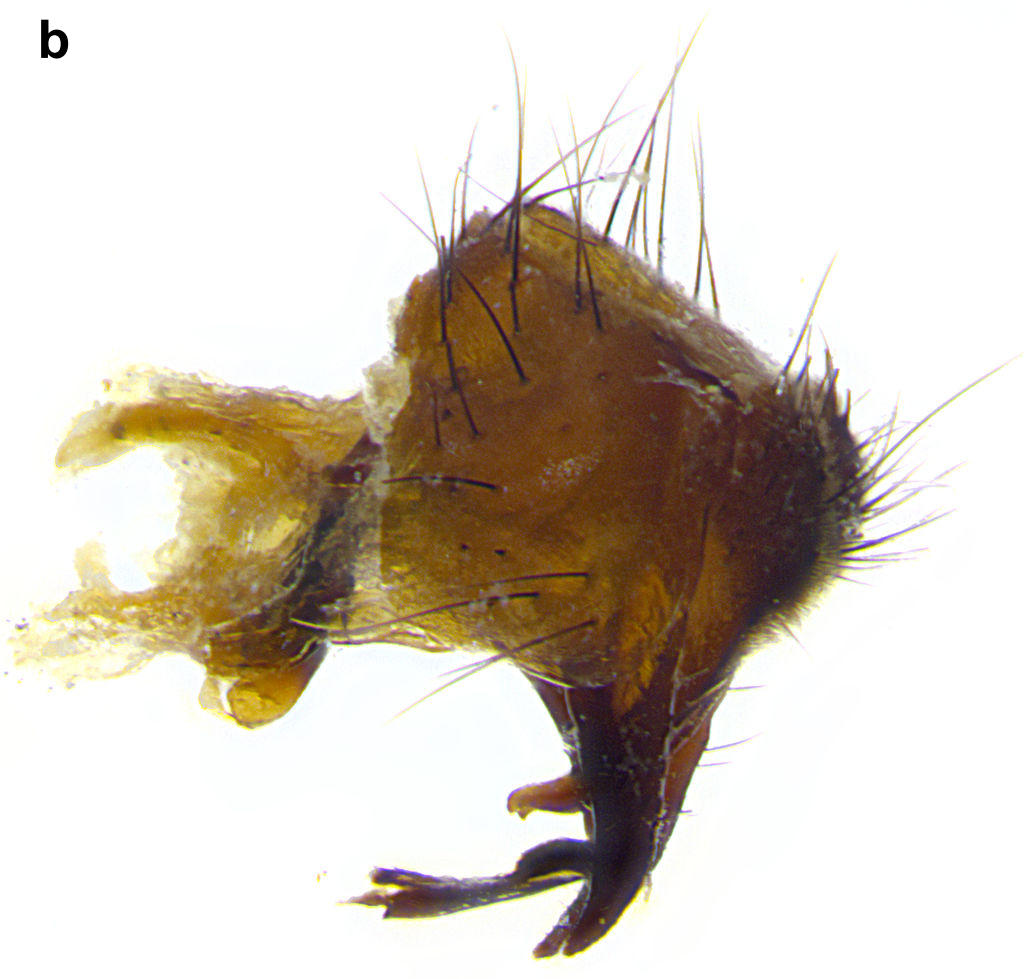
lateral view

**Figure 3c.**
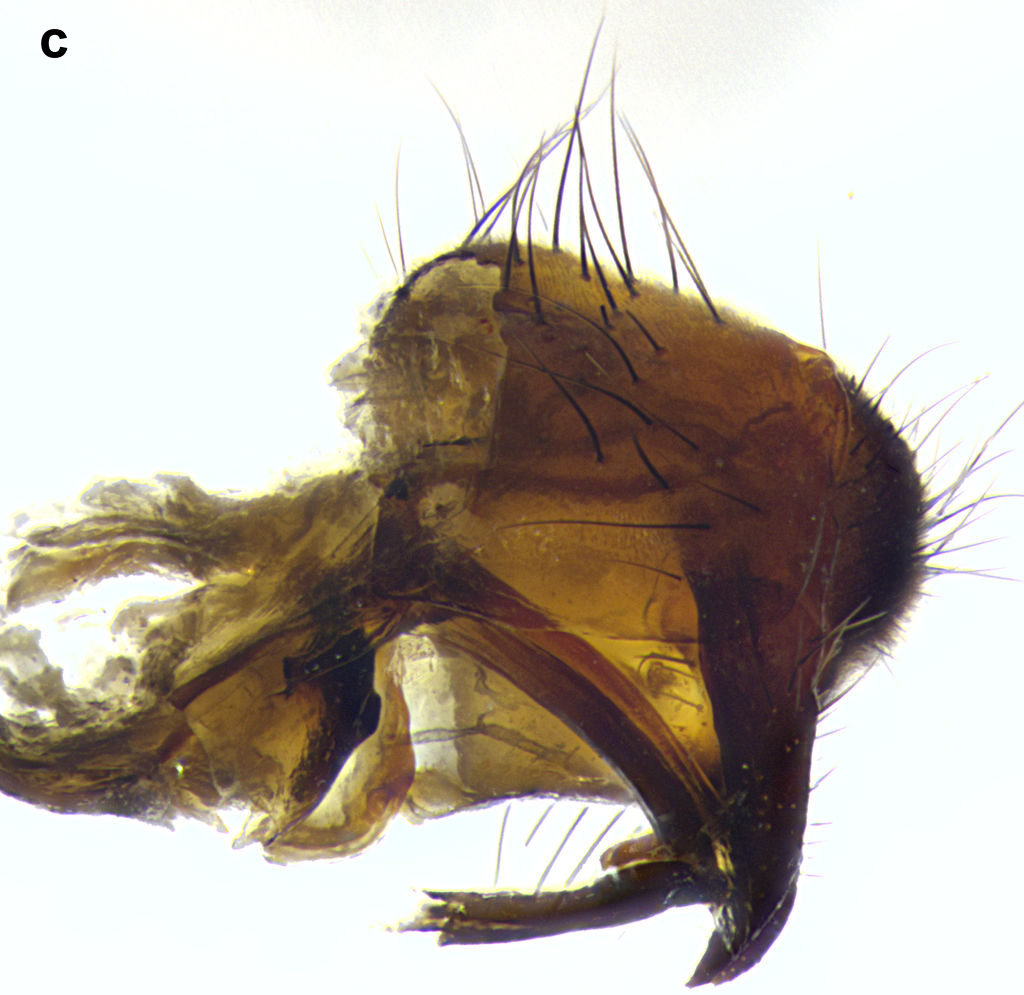
oblique lateral view

**Figure 3d.**
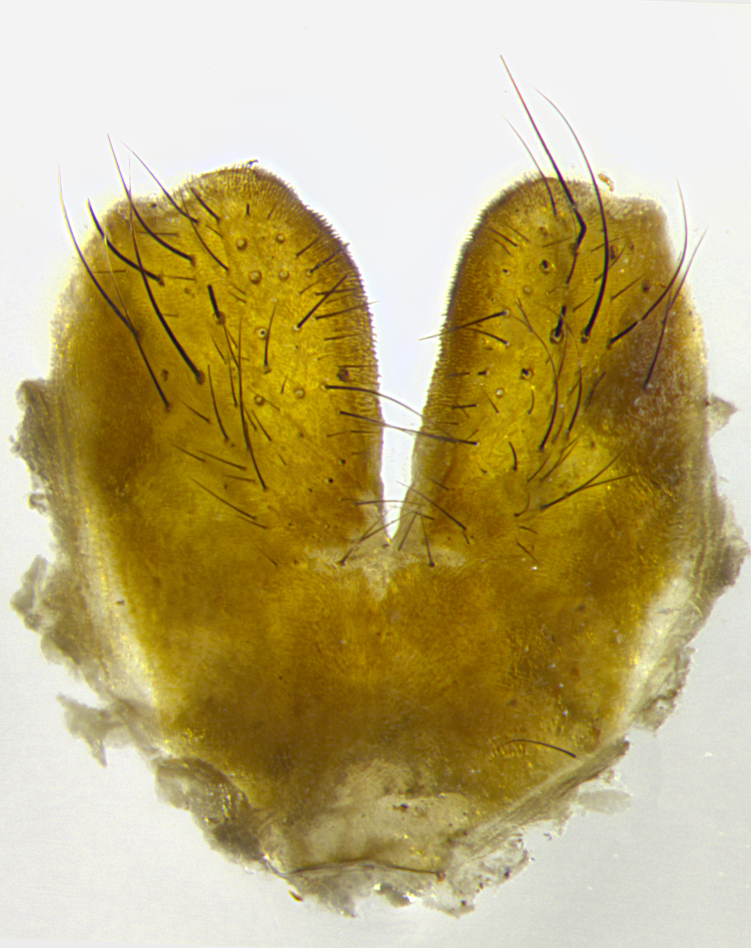
sternite 5, ventral view

**Figure 4a.**
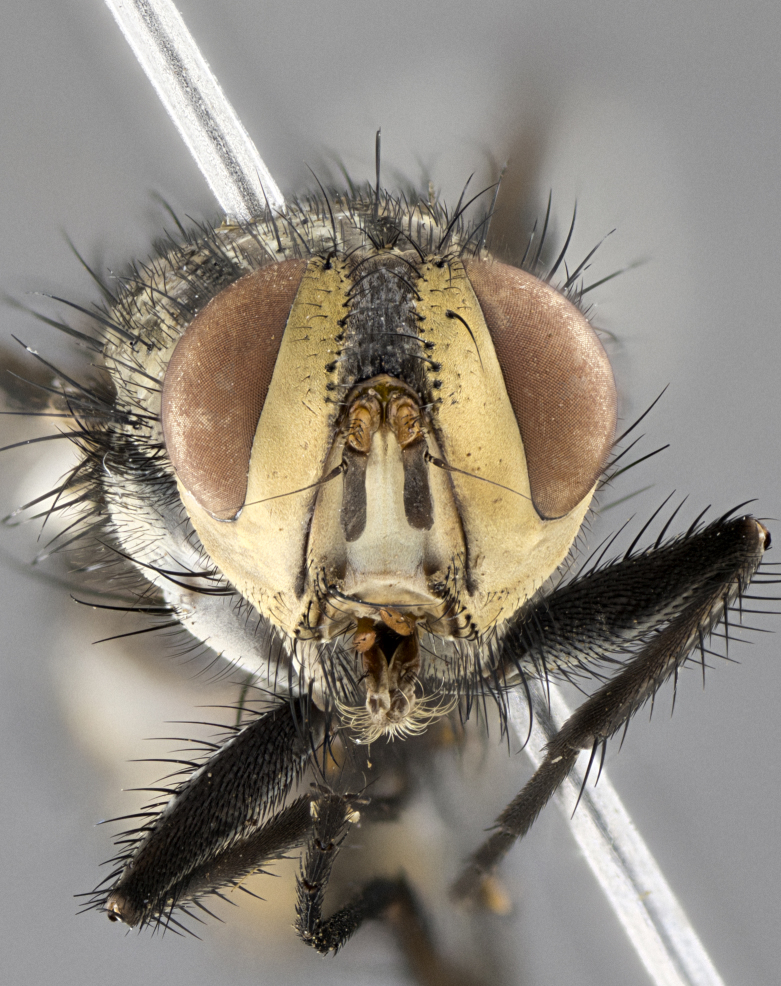
frontal view of head

**Figure 4b.**
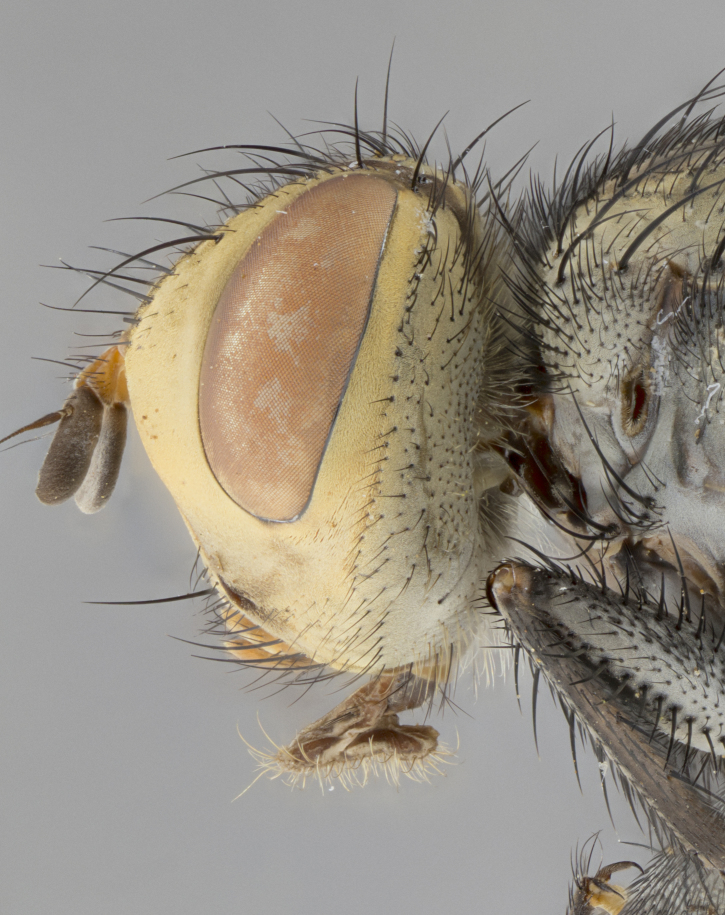
lateral view of head

**Figure 4c.**
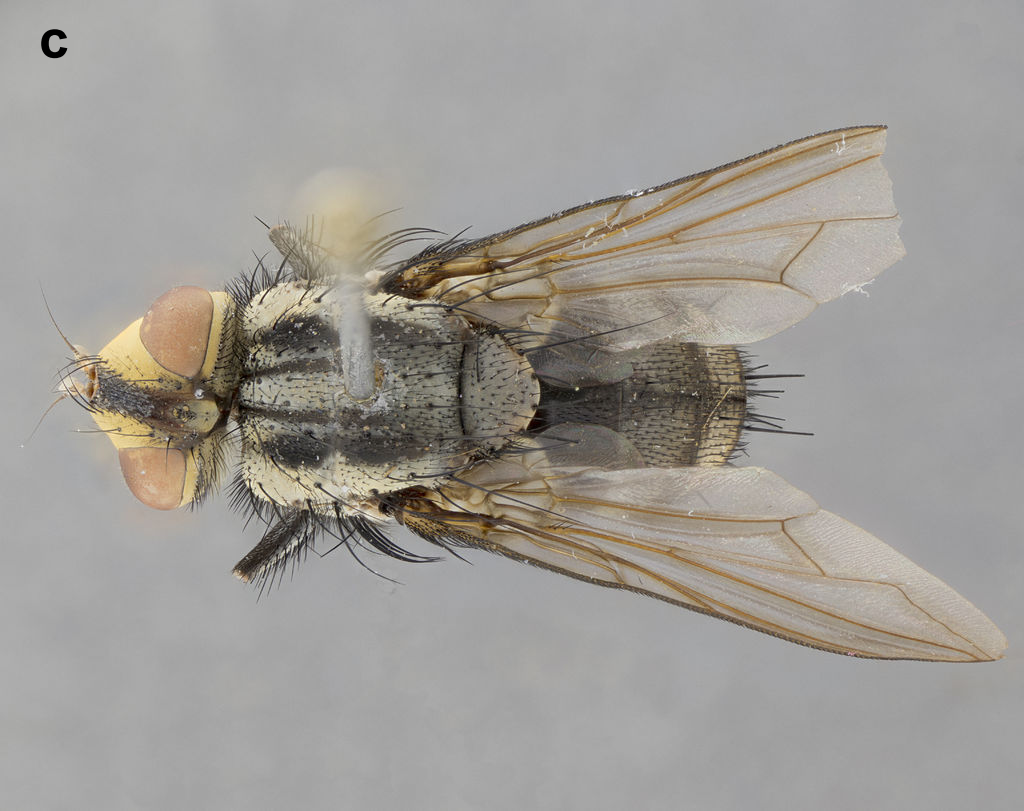
dorsal view

**Figure 4d.**
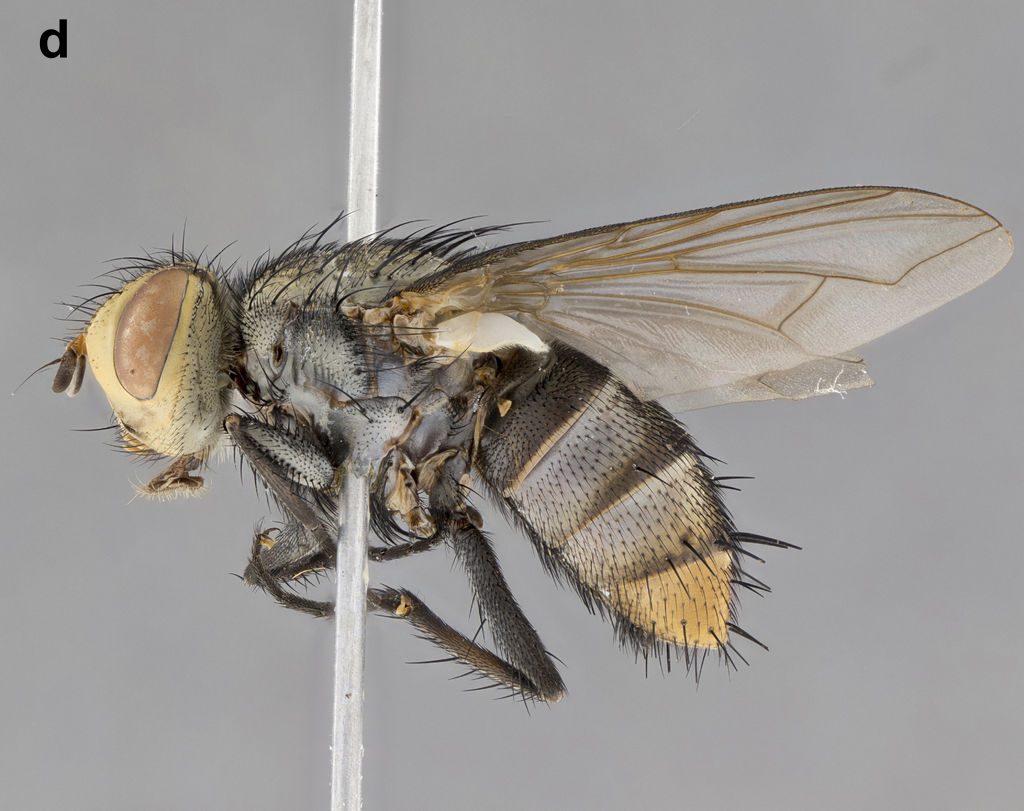
lateral view
